# Prognostic significance of the Naples prognostic score in colorectal cancer patients undergoing curative resection: a propensity score matching analysis

**DOI:** 10.1186/s12876-023-02722-6

**Published:** 2023-03-25

**Authors:** Atsushi Sugimoto, Tatsunari Fukuoka, Masatsune Shibutani, Hiroaki Kasashima, Kishu Kitayama, Masaichi Ohira, Kiyoshi Maeda

**Affiliations:** Department of Gastroenterological Surgery, Osaka Metropolitan University Graduate School of Medicine, 1-4-3 Asahimachi, Abeno-Ku, Osaka, 545-8585 Japan

**Keywords:** Naples prognostic score, Systemic inflammation, Colorectal cancer, Prognosis, Overall survival

## Abstract

**Background:**

Systemic inflammation is recognized as a hallmark of cancer that contributes to tumor development and progression in various malignancies. The Naples prognostic score (NPS) was established as a prognostic indicator for colorectal cancer (CRC). This study aims to examine the predictive value of the NPS for survival in CRC patients undergoing curative resection by a propensity score matching (PSM) analysis.

**Methods:**

A total of 533 CRC patients were enrolled in this study. Overall survival (OS) and disease-free survival (DFS) were compared between high-NPS and low-NPS groups. A time-dependent receiver operator characteristic (ROC) curve analysis was conducted to calculate the area under curve (AUC) of the NPS for OS. A multivariable Cox-proportional hazards regression analysis and PSM analysis were used to identify independent prognostic factors for OS and DFS. We compared the predictive value of the NPS to that of the neutrophil-to-lymphocyte ratio (NLR), lymphocyte-to-monocyte ratio (LMR), platelet-to-lymphocyte ratio (PLR), Onodera prognostic nutritional index (PNI), and controlling nutritional status score (CONUT) for OS.

**Results:**

High-NPS was significantly associated with worse OS and DFS. After PSM, 123 patients were included in each group. A multivariate analysis revealed that Age ≥ 68, ASA-PS ≥ 3, high NPS and undifferentiated tumor type were independently associated with OS, while high NPS, advanced T and N stage were independently associated with DFS after PSM. The NPS had the greatest AUC for OS in comparison to the NLR, LMR, PNI and CONUT.

**Conclusions:**

We successfully validated the prognostic utility of the NPS for CRC patients after curative resection.

## Introduction

Colorectal cancer (CRC) is ranking the second most common cause of cancer death [1]. Globally, surgical resection is currently the standard treatment for CRC without distant metastasis. Despite the development of surgical techniques and chemotherapy regimens for patients with CRC, the long-term outcomes of CRC remain unsatisfactory. TNM staging, which is a pathology-based system, has been widely used as a common risk assessment tool for predicting the prognosis in various types of cancer [2]. However, long-term survival would be different, even among patients with the same TNM stage. In order to improve the long-term survival of patients with CRC, robust prognostic biomarkers that can be used to identify high-risk patients could provide tremendous clinical benefits for individual postoperative follow-up plan and treatment strategy.

Systemic inflammation is a recognized as a hallmark of cancer that contributes to tumor development and progression in various type of cancer [3]. Current evidence indicates that the host systemic inflammatory and nutritional status are critical parameters predicting patient survival in CRC [4]. Previous studies have reported some inflammation-based and/or nutritional markers, such as the neutrophil-to-lymphocyte ratio (NLR), lymphocyte-to-monocyte ratio (LMR), platelet-to-lymphocyte ratio (PLR), Onodera prognostic nutritional index (PNI), and controlling nutritional status (CONUT) as predictive markers for long-term survival in CRC [5–9]. These markers are calculated based on the combination of the neutrophil, lymphocyte, monocyte, and platelet counts, serum albumin, and serum total cholesterol. The optimal scoring system for predicting the long-term survival of patients with CRC has not been identified.

The Naples prognostic score (NPS), which is based on serum albumin and serum total cholesterol, NLR, and LMR, was established by Galizia er al. as a predictive marker for the long-term survival of CRC patients [10]. Although the NPS has significant impact on survival in CRC, there would be a lot of difference in patients background factors, such as the presence of distant metastasis. In one study examining the utility of NPS in T1-2N0 CRC, the NPS was not correlated with both overall survival (OS) and disease-free survival (DFS) [11]. Thus, the confounding factors should be minimized to validate the predictive value of the NPS for survival of patients with CRC.

This study aimed to examine the predictive value of the NPS for survival in CRC patients undergoing curative resection. We compared the predictive value of the NPS to that of other inflammation-based and/or nutritional markers, including the NLR, LMR, PLR, PNI, and CONUT. To validate the utility of the NPS, we performed a propensity score matching (PSM) analysis.

## Material and methods

### Patients

The retrospective study enrolled consecutive patients who underwent curative surgical resection for CRC at the Department of Gastroenterological Surgery, Osaka Metropolitan University Hospital from January 2008 to December 2016. We excluded patients with pathological Stage 0 or IV, non-curative (R1 or R2) resection, neoadjuvant therapy (chemotherapy and/or radiotherapy), synchronous other cancer, and histologically atypical tumors, such as squamous cell carcinoma and neuroendocrine tumor. We reviewed the following clinical data from electronic medical records: age, sex, body mass index (BMI), Charlson comorbidity index (CCI) [12], American Society of Anesthesiologists classification of physical status (ASA-PS), serum albumin level, serum total cholesterol level, NPS, NLR, LMR, PLR, PNI, CONUT, tumor location (colon and rectum), histological tumor type (differentiated type; well- or moderately-differentiated adenocarcinoma and undifferentiated type; poorly-differentiated and mucinous type), pathological T (pT) stage, pathological N (pN) stage, pathological TNM stage (pStage) based on the 8^th^ edition of the Union for International Cancer Control TNM classification of malignant tumors [2], neoadjuvant treatment (chemotherapy, chemoradiotherapy or none), operative procedure (laparoscopy and open surgery). This study protocol was reviewed and approved by the Ethical Committee of Osaka Metropolitan University Graduate School of Medicine (approval number 926). Written informed consent was obtained from all participants. This study was conducted in according with the principles of the Declaration of Helsinki.

### Inflammation-based and/or nutritional markers

**Fig. 1:**
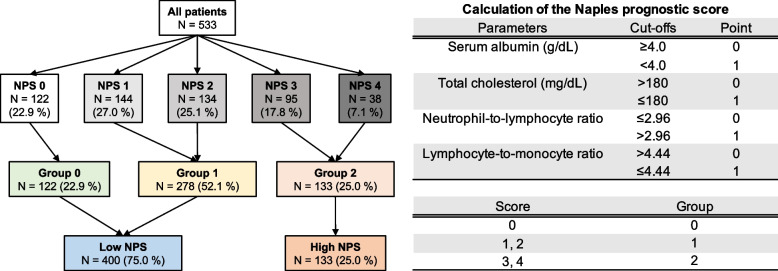
Flow chart of grouping patients and definition of Naples prognostic score (NPS)

A blood examination was performed before treatment in our hospital. The NPS was scored based on the following 4 parameters: serum albumin, total cholesterol, NLR and LMR, and divided into 3 groups based on the following scores: score 0, group 0; score 1–2, group 1; and score 3–4, group 2 defined as Fig. 1 [10]. The PNI was calculated as follows: 10 × albumin (g/dL) + 0.005 × TLC (defined by a previous report) [8]. The CONUT (including albumin: ≥ 3.5, 3.0 − 3.4, 2.5 − 2.9, < 2.5 g/dL; TLC: ≥ 1600, 1200 − 1599, 800 − 1199, < 800/μL and total cholesterol: ≥ 180, 140 − 179, 100 − 139, < 100 mg/dL) was calculated in accordance with the methods of a previous report [9]. All patients were classified into one of two groups based on the NPS (Low NPS, groups 0 and 1; high NPS, group 2).

### Treatment strategy

We treated CRC patients according to the Japanese Society for Cancer of the Colon and Rectum (JSCCR) guidelines [13]. Various radiological examinations were performed for the preoperative diagnosis, including colonoscopy and contrast-enhanced computed tomography (CT). Curative surgical resection was considered to have been achieved when a microscopic examination showed the absence of residual tumor cells at the stump of the surgical specimen with a sufficient surgical margin. Neoadjuvant chemotherapy (NAC) with capecitabine and/or oxaliplatin and neoadjuvant chemoradiotherapy (NACRT) with capecitabine and/or bevacizumab and radiotherapy (total dose, 50.4 Gy) were performed for patients with clinical T3/4 lower rectal cancer. All surgery was performed under general anesthesia (managed by anesthesiologists). Adjuvant chemotherapy was performed for patients with pStage II/III disease by monotherapy using an oral pro-drug based on 5-FU, such as capecitabine or combined therapy with 5-FU and oxaliplatin, such as 5-fluorouracil/leucovorin plus oxaliplatin (FOLFOX) or capecitabine plus oxaliplatin (CapeOX).

### Short- and long-term outcomes

We evaluated the short-term outcomes, including intraoperative blood loss, transfusion, operation time, overall complications, severe complications, and postoperative stay. Severe postoperative complications were defined as CDC grade ≥ III, which developed within 30 days after surgery. The long-term outcomes were analyzed based on the information in the electronic medical record. Patients were followed up at intervals of 3–6 months until the end of the study period or death. The OS and DFS were calculated from the start date of surgery to the date of last follow-up or death, and to the confirmed date of recurrence, respectively.

### Statistical analyses

Continuous variables are shown as the median (interquartile range [IQR]). The cut-off values of continuous variables were calculated by the time-dependent ROC curve for OS. PSM was performed to minimize the bias of baseline information. The matched baseline information was as follows: age, sex, BMI, CCI, tumor location, histological tumor type, pT and pN stage. Patients were matched 1:1 by the neighbor matching method via a caliper width with a standard deviation of 0.2. Univariate analyses were performed by the chi-squared test for categorical variables and by the Mann–Whitney U test for continuous variables. Survival probability (OS and DFS) was calculated by Kaplan–Meier survival curves and compared using the log-rank test. Univariate and multivariate analyses using a Cox proportional hazards model were performed to calculate hazard ratios (HRs) and 95% confidence intervals (CIs) and to identify significant prognostic factors for OS and DFS. Comparison of the predictive value of inflammation-based and/or nutritional markers was conducted based on AUCs calculated by the time-dependent ROC curve for OS. *P* values of < 0.05 were considered statistically significant. All statistical analyses were performed with EZR (Saitama Medical Center, Jichi Medical University, Saitama, Japan), which is a graphical user interface for R (The R Foundation for Statistical Computing, Vienna, Austria) [14].

## Results

### Patient characteristics

A total of 533 patients who underwent curative resection for CRC were included in this study. Table 1 summarized the clinical characteristics of the entire cohort. Figure 1 shows flow chart of grouping patients and definition of Naples prognostic score in this study. Before PSM, patients in NPS groups 0 and 1 (*n* = 400, 75.0%) were assigned to the low-NPS group, and those in NPS group 2 (*n* = 133, 25.0%) were assigned to the high-NPS group. After PSM, 127 patients were included in each group. According to the time-dependent ROC curve analysis for OS, the following cutoff values were set: age, 68 years; BMI, 18; CCI, 2; blood loss, 100 ml; operation time, 185 min.

**Table 1:** Clinical characteristics of all patients

		All patients
Characteristics		(*N* = 533)
Age	(years, IQR)	70 (63 − 76)
	< 68	218
	≥ 68	315
Sex	Male	313
	Female	220
BMI	(kg/m^2^, IQR)	22.4 (20.3 − 24.6)
	< 18	51
	≥ 18, < 25	369
	≥ 25	113
CCI	(IQR)	1 (0 − 2)
	0	247
	≥ 1	286
ASA-PS	< 3	458
	≥ 3	75
Albumin	(g/dL, IQR)	4.0 (3.7 − 4.3)
	< 4.0	235
	≥ 4.0	298
Total cholesterol	(mg/dL, IQR)	186 (163 − 212)
	≤ 180	224
	> 180	309
NLR	(IQR)	2.26 (1.73 − 3.21)
	≤ 2.96	372
	> 2.96	161
LMR	(IQR)	4.84 (3.37 − 6.17)
	≤ 4.44	232
	> 4.44	301
Tumor location	Colon	315
	Rectum	218
Histological type	Differentiated	511
	Undifferentiated	21
T stage	T1	154
	T2	87
	T3	228
	T4	64
N stage	N0	388
	N1	108
	N2	37
TNM stage	I	213
	II	175
	III	145
Surgical approach	Laparoscopy	394
	Open surgery	139

*NPS* Naples prognostic score, *BMI* Body mass index, *CCI* Charlson comorbidity index, *ASA-PS* The American Society of Anesthesiologists physical status, *NLR* Neutrophil-to-lymphocyte ratio, *LMR* Lymphocyte-to-monocyte ratio, *IQR* interquartile range

### Clinical characteristics of the high- and low-NPS groups

Table 2 summarizes the clinical characteristics of the high- and low-NPS groups before and after PSM. Before PSM, high-NPS was significantly associated with old age (*p* < 0.001), male sex (*p* = 0.033), low BMI (*p* = 0.047), high CCI (*p* < 0.001), high ASA-PS (*p* = 0.001), rectal cancer (*p* = 0.025), histological undifferentiated type (*p* = 0.008), advanced pT (*p* < 0.001), advanced pStage (*p* = 0.002), and open surgery (*p* = 0.023). After PSM, the clinical characteristics of the patients were well balanced.

**Table 2:** Clinical characteristics of the high- and low-NPS groups before and after propensity score matching

		Before matching				After matching			
		Low NPS	High NPS			Low NPS	High NPS		
Characteristics		(*N* = 400)	(*N* = 133)	*p* value	SD	(*N* = 123)	(*N* = 123)	*p* value	SD
Age	(years, IQR)	68 (62 − 75)	74 (68 − 80)	< 0.001	0.476	74 (70 − 79)	73 (67 − 80)	0.627	0.062
	< 68	186 (85.3%)	32 (14.7%)	< 0.001	0.483	24 (42.9%)	32 (57.1%)	0.287	0.156
	≥ 68	214 (67.9%)	101 (32.1%)			99 (52.1%)	91 (47.9%)		
Sex	Male	224 (71.6%)	89 (28.4%)	0.033	0.226	75 (48.4%)	80 (51.6%)	0.597	0.084
	Female	176 (80.0%)	44 (20.0%)			48 (52.7%)	43 (47.3%)		
BMI	(kg/m^2^, IQR)	22.5 (20.5 − 24.8)	22.1 (19.7 − 24.0)	0.047	0.2	22.4 (19.9 − 24.2)	22.1 (19.9 − 24.3)	0.945	0.009
	< 18	34 (66.7%)	17 (33.3%)	0.069	0.235	13 (48.1%)	14 (51.9%)	1	0.026
	≥ 18, < 25	273 (74.0%)	96 (26.0%)			90 (50.3%)	89 (49.7%)		
	≥ 25	93 (82.3%)	20 (17.7%)			20 (50.0%)	20 (50.0%)		
CCI	0	207 (83.8%)	40 (16.2%)	< 0.001	0.452	39 (50.6%)	38 (49.4%)	1	0.018
	≥ 1	193 (67.5%)	93 (32.5%)			84 (49.7%)	85 (50.3%)		
ASA-PS	< 3	356 (77.7%)	102 (22.3%)	0.001	0.331	102 (52.3%)	93 (47.7%)	0.208	0.181
	≥ 3	44 (58.7%)	31 (41.3%)			21 (41.2%)	30 (58.8%)		
Tumor location	Colon	225 (71.4%)	90 (28.6%)	0.025	0.237	81 (49.4%)	83 (50.6%)	0.892	0.034
	Rectum	175 (80.3%)	43 (19.7%)			42 (51.2%)	40 (48.8%)		
Histological type	Differentiated	389 (76.1%)	122 (23.9%)	0.008	0.257	117 (50.4%)	115 (49.6%)	0.784	0.07
	Undifferentiated	10 (47.6%)	11 (52.4%)			6 (42.9%)	8 (56.1%)		
T stage	T1	132 (85.7%)	22 (14.3%)	< 0.001	0.471	21 (48.8%)	22 (51.2%)	0.911	0.097
	T2	69 (79.3%)	18 (20.7%)			22 (55.0%)	18 (45.0%)		
	T3	161 (70.6%)	67 (29.4%)			57 (48.3%)	61 (51.7%)		
	T4	38 (59.3%)	26 (40.7%)			23 (51.1%)	22 (48.9%)		
N stage	N0	298 (76.8%)	90 (23.2%)	0.285	0.151	89 (51.1%)	85 (48.9%)	0.858	0.072
	N1	76 (70.4%)	32 (29.6%)			27 (47.4%)	30 (52.6%)		
	N2	26 (70.3%)	11 (29.7%)			7 (46.7%)	8 (53.3%)		
TNM stage	I	177 (83.1%)	36 (16.9%)	0.002	0.365	42 (53.8%)	36 (46.2%)	0.696	0.109
	II	121 (69.1%)	54 (30.9%)			47 (49.0%)	49 (51.0%)		
	III	102 (70.3%)	43 (29.7%)			34 (47.2%)	38 (52.8%)		
Surgical approach	Laparoscopy	306 (77.7%)	88 (22.3%)	0.023	0.23	90 (52.6%)	81 (47.4%)	0.268	0.159
	Open surgery	94 (67.6%)	45 (32.4%)			33 (44.0%)	42 (56.0%)		

*NPS* Naples prognostic score, *SD* standardized difference, *BMI* Body mass index, *CCI* Charlson comorbidity index, *ASA-PS* The American Society of Anesthesiologists physical status, *IQR* interquartile range

### Short-term outcomes

Before PSM, high-NPS was significantly associated with large intraoperative blood loss (*p* = 0.027) and transfusion (*p* = 0.003) (Table 3). After PSM, high-NPS was significantly associated with transfusion (*p* = 0.033).

**Table 3:** Short-term outcomes of the high- and low-NPS groups before and after propensity score matching

			Before matching			After matching		
		All patients	Low NPS	High NPS		Low NPS	High NPS	
Outcomes		(*N* = 533)	(*N* = 400)	(*N* = 133)	*p* value	(*N* = 123)	(*N* = 123)	*p* value
Blood loss	(mL, IQR)	40 (20 − 140)	40 (20 − 106)	50 (20 − 260)	0.027	50 (20 − 120)	50 (18 − 235)	0.391
	< 100	364	282 (77.5%)	82 (22.5%)	0.067	82 (52.2%)	75 (47.8%)	0.426
	≥ 100	169	118 (69.8%)	51 (30.2%)		41 (46.1%)	48 (53.9%)	
Transfusion	Yes	33	17 (51.5%)	16 (48.5%)	0.003	5 (25.0%)	15 (75.0%)	0.033
	No	500	383 (76.6%)	117 (23.4%)		118 (52.2%)	108 (47.8%)	
Operation time	(min, IQR)	221 (180 − 273)	217 (176 − 274)	225 (191 − 263)	0.426	214 (173 − 271)	223 (192 − 261)	0.376
	< 185	149	121 (81.2%)	28 (18.8%)	0.045	38 (60.3%)	25 (39.5%)	0.079
	≥ 185	384	279 (72.7%)	105 (27.3%)		85 (46.4%)	98 (53.6%)	
Overall complications	Yes	219	159 (72.6%)	60 (27.4%)	0.309	52 (48.1%)	56 (51.9%)	0.7
	No	314	241 (76.8%)	73 (23.2%)		71 (51.4%)	67 (48.6%)	
Severe complications	Yes	88	60 (68.2%)	28 (31.8%)	0.107	16 (37.2%)	27 (62.8%)	0.237
	No	445	340 (76.4%)	105 (23.6%)		107 (52.7%)	96 (47.3%)	
Postoperative stay	(days, IQR)	12 (10 − 18)	12 (10 − 17)	13 (11 − 19)	0.137	12 (10 − 17)	13 (11 − 20)	0.241
Adjuvant chemotherapy	Yes	172	131 (76.2%)	41 (23.8%)	0.748	46 (56.1%)	36 (43.9%)	0.223
	No	361	269 (74.5%)	92 (25.5%)		77 (47.0%)	87 (53.0%)	

*NPS* Naples prognostic score, *IQR* interquartile range

### Long-term outcomes

**Fig. 2:**
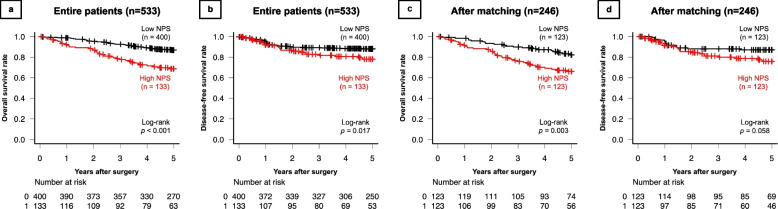
Kaplan–Meier survival analyses of the high- and low-NPS groups in the overall study population (*n* = 533). **a** Overall survival (OS). The OS rate in the high-NPS group was significantly lower than that in the low-NPS group (*p* < 0.001). **b** The disease-free survival (DFS). The DFS rate in the high-NPS group was significantly lower than that in the low-NPS group (*p* = 0.017). **c** OS after propensity score matching. The OS rate in the high-NPS group was significantly lower than that in the low-NPS group after matching (*p* = 0.003). **d** DFS after propensity score matching. The DFS rates of the high and low NPS groups (≥ 7) did not differ to a statistically significant extent (*p* = 0.058)

**Fig. 3:**
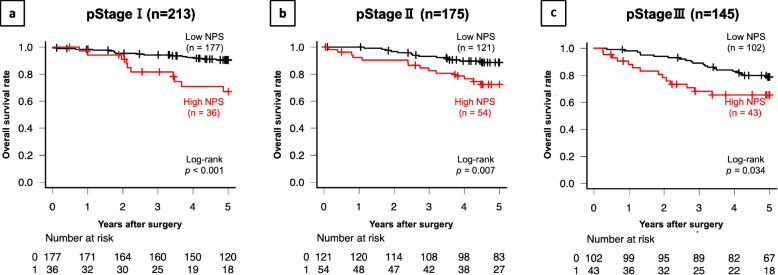
Overall survival (OS) according to pStage. **a** OS in 213 patients with pStage I. The OS rate in the high-NPS group was significantly lower than that in the low-NPS group after propensity score matching (*p* < 0.001). **b** OS in 175 patients with pStage II disease. The OS rate in the high-NPS group was significantly lower than that in the low-NPS group after propensity score matching (*p* = 0.007). **c** OS in 145 patients with pStage III. The OS rate in the high-NPS group was significantly lower than that in the low-NPS group after propensity score matching (*p* = 0.034)

The median follow-up time was 63 (IQR, 51 − 75) months. The 5-year OS and DFS rates for the overall population were 82.6% and 85.9%, respectively. After PSM, the 5-year OS and DFS rates for the matched patients were 74.4% and 81.9%, respectively. Figure 2 shows Kaplan–Meier survival curves comparing OS and DFS between the two groups. Before PSM, the OS and DFS rates in the high-NPS group were significantly lower than those in the low-NPS group (*p* < 0.001 and *p* = 0.017, respectively) (Fig. 2a, b). Furthermore, Fig. 3 shows that The OS rates in the high-NPS group were significantly lower than those in the low-NPS group among patients with pStage I, II and III disease (*p* < 0.001, *p* = 0.007 and *p* = 0.034 respectively). After PSM, the OS in the high-NPS group was significantly lower in comparison to the low-NPS group (*p* = 0.003) (Fig. 2c, d).

### Univariate and multivariate analyses for OS and DFS

Table 4 shows the univariate and multivariate analyses for OS and DFS before and after PSM. Before PSM, univariate analyses revealed that age ≥ 68 years, male sex, CCI ≥ 1, ASA-PS ≥ 3, high-NPS, histological undifferentiated type, pT ≥ 3, pN positivity, pStage III, open surgery and blood loss ≥ 100 mL were significantly associated with a low OS rate. In the multivariate analysis identified age ≥ 68 years, ASA-PS ≥ 3, high-NPS, histological undifferentiated type and pN positivity showed an independent association with OS. On the other hand, in the univariate analyses, high-NPS, pT ≥ 3, pN positivity, pStage III, open surgery, blood loss ≥ 100 mL, transfusion, operative time ≥ 185 min, and adjuvant chemotherapy were significantly associated with a low DFS rate. In the multivariate analysis, pT ≥ 3, pN positivity, open surgery, and operative time ≥ 185 min showed an independent association with DFS. After PSM, univariate analyses revealed that age ≥ 68 years, ASA-PS ≥ 3, high-NPS, and undifferentiated tumor type were significantly associated with low OS rate. In the multivariate analysis, ASA-PS ≥ 3 and high-NPS showed an independent association with OS. In contrast, in the univariate analyses, pT ≥ 3, pN positivity, pStage III, and adjuvant chemotherapy were found to be significantly associated with low DFS. In the multivariate analysis, high-NPS, pT ≥ 3 and pN positivity showed an independent association with DFS.

**Table 4:** Univariate and multivariate analyses for OS and DFS before and after propensity score matching

	Analysis for OS (before matching)				Analysis for OS (after matching)			
	Univariate analysis		Multivariate analysis		Univariate analysis		Multivariate analysis	
	HR (95% CI)	*p* value	HR (95% CI)	*p* value	HR (95% CI)	*p* value	HR (95% CI)	*p* value
Age ≥ 68 years (vs < 68 years)	2.38 (1.47 − 3.86)	< 0.001	1.75 (1.07 − 2.89)	0.027	3.02 (1.30 − 7.04)	0.01	3.45 (1.45 − 8.24)	0.005
Male (vs Female)	1.69 (1.08 − 2.66)	0.023	1.58 (0.99 − 2.51)	0.054	1.44 (0.83 − 2.51)	0.199		
BMI ≥ 18 kg/m2 (vs < 18 kg/m2)	1.43 (0.62 − 3.28)	0.395			1.05 (0.45 − 2.44)	0.913		
CCI ≥ 1 (vs < 1)	1.96 (1.26 − 3.06)	0.003	1.22 (0.75 − 1.98)	0.413	1.82 (0.98 − 3.38)	0.057	1.74 (0.89 − 3.41)	0.105
ASA-PS ≥ 3 (vs < 3)	4.29 (2.76 − 6.67)	< 0.001	3.42 (2.11 − 5.54)	< 0.001	3.25 (1.92 − 5.50)	< 0.001	2.25 (1.27 − 3.98)	0.005
High NPS (vs Low NPS)	2.76 (1.81 − 4.21)	< 0.001	1.69 (1.08 − 2.64)	0.021	2.23 (1.30 − 3.84)	0.004	2.23 (1.27 − 3.89)	0.005
Rectum (vs Colon)	0.83 (0.54 − 1.28)	0.393			0.66 (0.37 − 1.19)	0.166		
Undifferentiated (vs Differentiated)	4.34 (2.24 − 8.38)	< 0.001	3.22 (1.61 − 6.43)	< 0.001	3.59 (1.70 − 7.58)	< 0.001	5.32 (2.28 − 12.4)	< 0.001
T stage ≥ 3 (vs < 2)	1.85 (1.18 − 2.89)	0.007	1.07 (0.65 − 1.76)	0.789	1.12 (0.64 − 1.96)	0.688		
N stage ≥ 1 (vs 0)	1.87 (1.22 − 2.87)	0.004	1.82 (1.14 − 2.88)	0.011	1.42 (0.83 − 2.42)	0.204		
Stage III (vs I and II)	1.87 (1.22 − 2.87)	0.004			1.42 (0.83 − 2.42)	0.204		
Open Surgery (vs Laparoscopy)	2.30 (1.51 − 3.51)	< 0.001	1.54 (0.91 − 2.62)	0.109	1.58 (0.94 − 2.68)	0.087	1.26 (0.72 − 2.21)	0.42
Blood loss ≥ 100 mL (vs < 100 mL)	1.88 (1.23 − 2.86)	0.003	1.32 (0.78 − 2.22)	0.299	1.41 (0.84 − 2.36)	0.2		
Transfusion	1.42 (0.66 − 3.07)	0.374			0.85 (0.31 − 2.35)	0.754		
Operation time ≥ 185 min (vs < 185 min)	1.04 (0.65 − 1.66)	0.873			0.69 (0.40 − 1.19)	0.177		
Severe complications	1.51 (0.91 − 2.50)	0.115			1.51 (0.81 − 2.79)	0.193		
Adjuvant chemotherapy	0.91 (0.58 − 1.42)	0.674			0.54 (0.30 − 0.99)	0.046	0.63 (0.33 − 1.23)	0.17
	Analysis for DFS (before matching)				Analysis for DFS (after matching)			
	Univariate analysis		Multivariate analysis		Univariate analysis		Multivariate analysis	
	HR (95% CI)	*p* value	HR (95% CI)	*p* value	HR (95% CI)	*p* value	HR (95% CI)	*p* value
Age ≥ 68 years (vs < 68 years)	1.28 (0.79 − 2.08)	0.32			1.28 (0.59 − 2.79)	0.531		
Male (vs Female)	1.45 (0.89 − 2.38)	0.14			1.05 (0.55 − 2.00)	0.884		
BMI ≥ 18 kg/m2 (vs < 18 kg/m2)	1.76 (0.64 − 4.84)	0.27			2.27 (0.55 − 9.41)	0.26		
CCI ≥ 1 (vs < 1)	1.11 (0.69 − 1.77)	0.669			0.79 (0.41 − 1.51)	0.477		
ASA-PS ≥ 3 (vs < 3)	0.97 (0.47 − 2.04)	0.944			0.53 (0.19 − 1.50)	0.235		
High NPS (vs Low NPS)	1.81 (1.11 − 2.97)	0.018	1.26 (0.75 − 2.11)	0.377	1.85 (0.97 − 3.53)	0.062	1.97 (1.02 − 3.78)	0.043
Rectum (vs Colon)	1.33 (0.83 − 2.12)	0.239			0.95 (0.49 − 1.85)	0.884		
Undifferentiated (vs Differentiated)	1.87 (0.68 − 5.12)	0.227			1.88 (0.58 − 6.13)	0.294		
T stage ≥ 3 (vs < 2)	5.54 (2.84 − 10.8)	< 0.001	3.26 (1.59 − 6.67)	0.001	4.91 (1.75 − 13.8)	0.003	3.07 (1.02 − 9.27)	0.047
N stage ≥ 1 (vs 0)	4.59 (2.84 − 7.40)	< 0.001	3.15 (1.77 − 5.61)	< 0.001	4.56 (2.39 − 8.69)	< 0.001	3.44 (1.59 − 7.34)	0.002
Stage III (vs I and II)	4.59 (2.84 − 7.40)	< 0.001			4.56 (2.39 − 8.69)	< 0.001		
Open Surgery (vs Laparoscopy)	2.28 (1.42 − 3.67)	< 0.001	2.11 (1.12 − 3.97)	0.02	1.71 (0.90 − 3.23)	0.1		
Blood loss ≥ 100 mL (vs < 100 mL)	1.78 (1.11 − 2.86)	0.017	0.75 (0.40 − 1.43)	0.385	1.30 (0.69 − 2.46)	0.421		
Transfusion	2.49 (1.24 − 5.01)	0.011	1.48 (0.71 − 3.10)	0.296	1.76 (0.69 − 4.49)	0.239		
Operation time ≥ 185 min (vs < 185 min)	1.94 (1.04 − 3.61)	0.037	1.95 (1.01 − 3.77)	0.046	1.35 (0.62 − 2.93)	0.454		
Severe complications	1.39 (0.77 − 2.50)	0.271			1.40 (0.64 − 3.05)	0.394		
Adjuvant chemotherapy	3.38 (2.09 − 5.47)	< 0.001	1.08 (0.60 − 1.94)	0.809	2.68 (1.42 − 5.07)	0.002	0.99 (0.45 − 2.14)	0.97

*OS* Overall survival, *DFS* Disease free survival, *BMI* Body mass index, *CCI* Charlson comorbidity index, *ASA-PS* The American Society of Anesthesiologists physical status, *NPS* Naples prognostic score, *HR* Hazard ratio, *CI* confidence interval

### Comparison of the predictive value of inflammation-based and/or nutritional markers

**Fig. 4:**
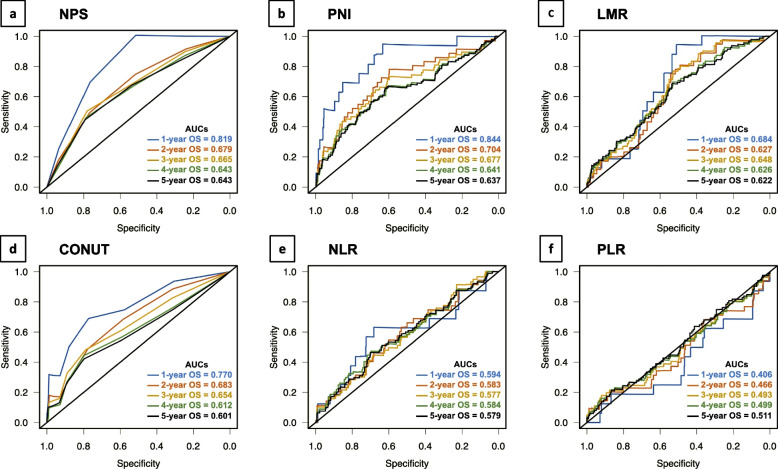
Time-dependent ROC curve-analyses for overall survival. **a** Naples prognostic score (NPS), **b** Onodera prognostic nutritional index (PNI), **c** lymphocyte to monocyte ratio (LMR), **d** controlling nutritional status score (CONUT), **e** neutrophil to lymphocyte ratio (NLR), **f** platelet to lymphocyte ratio (PLR)

Figure 4 indicates that NPS have the greatest AUC of 5-year OS (AUC = 0.643) in the inflammation-based and/or nutritional markers of the entire cohort. The AUCs of 5-year OS for PNI, LMR, CONUT, NLR, and PLR were 0.637, 0.622, 0.601, 0.579, and 0.511, respectively.

## Discussion and conclusions

In the present study, we examined the predictive value of the NPS on the prognosis after curative surgery for CRC without distant metastasis. High-NPS was significantly associated with a poor prognosis at each pStage. We revealed that high-NPS was an independent predictor of OS and DFS in CRC both before and after PSM. In comparison to other inflammation-based and/or nutritional markers, including the PNI, LMR, CONUT, NLR and PLR, the NPS had the greatest AUC for 5-year OS in the time-dependent ROC analysis. Our findings suggested that the NPS may be a helpful prognostic indicator for CRC patients who have undergone curative surgery.

Previous studies reported that the NPS was associated with long-term outcomes in CRC patients [10, 15]. However, these studies included CRC patients with metastatic disease, which may have contributed to many differences in baseline characteristics, which would have considerably affected the prognostic value of the NPS. To minimize the selection bias, this study enrolled CRC patients with radical surgery. In the present study, after PSM, high-NPS was significantly associated with transfusion. After PSM, ASA-PS was found to be independently associated with OS in CRC patients, and advanced T and N stage were found to be independently associated with DFS in CRC patients. These results are consistent with previous studies [16, 17]. Furthermore, after PSM, the multivariate analysis revealed that NPS was an independent prognostic factor for OS and DFS in CRC patients. To our knowledge, this is the first study to successfully validate the prognostic significance of the NPS for OS and DFS in CRC using a PSM analysis.

Serum albumin and serum total cholesterol have been used as nutritional and systemic inflammation markers predicting a worse prognosis in gastrointestinal cancer [18, 19]. Systematic inflammation markers such as the NLR, LMR, and PLR have been reported as significant prognostic markers in CRC [5, 6, 20]. However, the prognostic value of a single inflammation or nutrition marker has been insufficient. Recently, combined scoring systems such as the PNI and CONUT, which are calculated using serum albumin, the lymphocyte count and total cholesterol, have been reported as prognostic markers in CRC [8, 9]. However, the optimal prognostic marker for CRC has been unclear. In the present study, the time-dependent ROC analysis revealed that the NPS provided the highest AUC value for predicting 5-year OS in CRC in comparison to other inflammation-based and/or nutritional markers, including the PNI, LMR, CONUT, NLR and PLR.

The biological mechanism through which the NPS contributes to the prognosis after surgery for CRC could be explained as follows. The NPS is composed of serum albumin and serum total cholesterol, NLR, and LMR. Serum albumin reflect the systemic inflammatory response to cancer [18]. Serum total cholesterol is associated with anti-cancer immunity, such as the activation of circulating lymphocytes [19, 21]. Immune cells mediate the cancer-associated inflammation and anti-cancer immune response in cancer patients [22]. The number of neutrophils, lymphocytes, and monocytes was associated with survival of CRC [23–25]. Tumor-associated neutrophils (TANs), macrophages (TAMs), and tumor infiltrating lymphocytes (TILs) are essential for facilitating cancer progression through the interaction of immune and cancer cells within the tumor microenvironment, [26, 27]. Thus, the NPS may be a useful assessment tool that could reflect cancer-associated inflammation and the immune response to cancer in CRC patients.

TNM staging is a common prognostic assessment system [2]. However, even patients with the same TNM stage may show different clinical outcomes. Despite the favorable prognosis that has generally been reported for early-stage CRC without lymph node metastases,　some patients with risk factors show poor outcomes. [28]. According to a previous study, the NPS was not significantly associated with the prognosis in early-stage CRC [11]. TNM staging cannot accurately predict the benefit of adjuvant chemotherapy in advanced-stage CRC with lymph node metastasis [29]. We suggested that the prognostic value of the NPS should be assessed separately at each stage. Our subgroup analysis indicated that the NPS was significantly associated with the OS of CRC patients with pStage I, II and III disease. Our findings suggested that the management of follow-up and the personalization of adjuvant chemotherapy for CRC in each stage based on NPS may lead favorable clinical outcomes.

The present study was associated with several limitations. Firstly, this was a single-center retrospective cohort study. Although we could successfully validate the feasibility of the NPS using PSM as internal validation, several selection biases could not be ignored. To eliminate any potential confounding factors, further large-scale external validation is warranted. Secondly, this study focused on prognostic implications of the NPS in patients with CRC who underwent curative resection. Although these patients were treated with heterogeneous chemotherapy, the clinical data were insufficient. Thirdly, this study did not include data on the genetic status (e.g., KRAS, BRAF mutations and microsatellite instability [MSI]), which strongly influence the prognosis of patients with CRC. Finally, this study solely enrolled patients of Japanese origin. Prospective studies that include diverse ethnic populations would be necessary for additional validation.

## Conclusion

High-NPS was an independent prognostic factor for OS and DFS in CRC patients undergoing curative resection. Our findings suggest that the NPS may be a helpful prognostic indicator for CRC patients who have undergone curative surgery.

## Data Availability

The datasets generated and/or analyzed during the current study are not publicly available due to confidentiality of information but are available from the corresponding author on reasonable request.
